# Supplementary material for: Using electronic health records to streamline provider recruitment for implementation science studies
Source: PLoS One. 2022 May 13;17(5):e0267915. doi: 10.1371/journal.pone.0267915 (PMC9106149; doi:10.1371/journal.pone.0267915)
Supplement: S1 Appendix — Bladder Cancer diagnosis based on the International Classification of Diseases, Ninth and Tenth Revision diagnosis codes (ICD-9, ICD-10). (DOCX) [file pone.0267915.s001.docx]

# APPENDIX.

Cystoscopy procedures based on International Classification of Diseases, Ninth and Tenth Revision procedure codes and Common Procedural Terminology (CPT) codes. Bladder Cancer diagnosis based on the International Classification of Diseases, Ninth and Tenth Revision diagnosis codes (ICD-9, ICD-10).

A: Cystoscopy CPT Codes

| **CPT Code** | **CPT Description** |
| --- | --- |
|  | **Cystoscopy with or without irrigation, urethral catheterization, or biopsy** |
| **52000** | Cystourethroscopy (separate procedure) |
| **52001** | Cystourethroscopy with irrigation and evacuation of multiple obstructing clots |
| **52005** | Cystourethroscopy, with ureteral catheterization, with or without irrigation, instillation, or ureteropyelography, exclusive of radiologic service; |
| **52007** | Cystourethroscopy, with ureteral catheterization, with or without irrigation, instillation, or ureteropyelography, exclusive of radiologic |
| **52204** | Cystourethroscopy, with biopsy(s) |
| **52214** | Cystourethroscopy, with fulguration (including cryosurgery or laser surgery) of trigone, bladder neck, prostatic fossa, urethra, or periurethral glands |
| **Cystoscopy with urethral dilation or urethrotomy/meatotomy** | |
| **52270** | Cystourethroscopy, with internal urethrotomy; female |
| **52275** | Cystourethroscopy, with internal urethrotomy; male |
| **52276** | Cystourethroscopy with direct vision internal urethrotomy |
| **52277** | Cystourethroscopy, with resection of external sphincter (sphincterotomy) |
| **52281** | Cystourethroscopy, with calibration and/or dilation of urethral stricture or stenosis, with or without meatotomy, with or without injection procedure for cystography, male or female |
| **52282** | Cystourethroscopy, with insertion of permanent urethral stent |
| **52283** | Cystourethroscopy, with steroid injection into stricture |
| **52285** | Cystourethroscopy for treatment of the female urethral syndrome with any or all the following: urethral meatotomy, urethral dilation, internal urethrotomy, lysis of urethrovaginal septal fibrosis, lateral incisions of the bladder neck, and fulguration of polyp(s) of urethra, bladder neck, and/or trigone |
| **52290** | Cystourethroscopy; with ureteral meatotomy, unilateral or bilateral |
| **52300** | Cystourethroscopy; with resection or fulguration of orthotopic ureterocele(s), unilateral or bilateral |
| **52301** | Cystourethroscopy; with resection or fulguration of ectopic ureterocele(s), unilateral or bilateral |
| **52305** | Cystourethroscopy; with incision or resection of orifice of bladder diverticulum, single or multiple |

B: ICD9 procedure codes

| **ICD9 Procedure Code** | **ICD9 Procedure Description** |
| --- | --- |
| **Cystoscopy with or without other intervention** | |
| **57.00** | Transurethral clearance of bladder |
| **57.32** | Other cystoscopy |
| **57.39** | Other diagnostic procedures on bladder |
| **Cystoscopy with biopsy / fulguration** | |
| **57.33** | Closed (transurethral) biopsy of bladder |
| **Cystoscopy with dilation** | |
| **57.91** | Sphincterotomy of bladder |
| **57.92** | Dilation of bladder neck |
|  | |

C: ICD10 procedure codes

| **ICD10 Procedure Code** | **ICD10 Procedure Description** |
| --- | --- |
| **Cystoscopy with dilation** | |
| **0T7C7DZ** | Dilation of Bladder Neck with Intraluminal Device, Via Natural or Artificial Opening |
| **0T7C7ZZ** | Dilation of Bladder Neck, Via Natural or Artificial Opening |
| **0T7C8DZ** | Dilation of Bladder Neck with Intraluminal Device, Via Natural or Artificial Opening Endoscopic |
| **0T7C8ZZ** | Dilation of Bladder Neck, Via Natural or Artificial Opening Endoscopic |
| **0T7D7DZ** | Dilation of Urethra with Intraluminal Device, Via Natural or Artificial Opening |
| **0T7D7ZZ** | Dilation of Urethra, Via Natural or Artificial Opening |
| **0T7D8DZ** | Dilation of Urethra with Intraluminal Device, Via Natural or Artificial Opening Endoscopic |
| **0T7D8ZZ** | Dilation of Urethra, Via Natural or Artificial Opening Endoscopic |
| **Cystoscopy with biopsy / fulguration** | |
| **0TBB7ZX** | Excision of Bladder, Via Natural or Artificial Opening, Diagnostic |
| **0TBB7ZZ** | Excision of Bladder, Via Natural or Artificial Opening |
| **0TBB8ZX** | Excision of Bladder, Via Natural or Artificial Opening Endoscopic, Diagnostic |
| **0TBB8ZZ** | Excision of Bladder, Via Natural or Artificial Opening Endoscopic |
| **Cystoscopy with or without other intervention** | |
| **0TJB7ZZ** | Inspection of Bladder, Via Natural or Artificial Opening |
| **0TJB8ZZ** | Inspection of Bladder, Via Natural or Artificial Opening Endoscopic |
|  | |

D: Bladder Cancer ICD9 and ICD10 Codes

| **ICD9 Code** | **ICD9 Description** |
| --- | --- |
| **188.0** | Malignant neoplasm of trigone of urinary bladder |
| **188.1** | Malignant neoplasm of dome of urinary bladder |
| **188.2** | Malignant neoplasm of lateral wall of urinary bladder |
| **188.3** | Malignant neoplasm of anterior wall of urinary bladder |
| **188.4** | Malignant neoplasm of posterior wall of urinary bladder |
| **188.5** | Malignant neoplasm of bladder neck |
| **188.6** | Malignant neoplasm of ureteric orifice |
| **188.7** | Malignant neoplasm of urachus |
| **188.8** | Malignant neoplasm of other specified sites of bladder |
| **188.9** | Malignant neoplasm of bladder, part unspecified |
| **233.7** | Carcinoma in situ of bladder |
| **236.7** | Neoplasm of uncertain behavior of bladder |
| **239.4** | Neoplasm of unspecified nature of bladder |
| **V10.50** | Personal history of malignant neoplasm of unspecified urinary organ |
| **V10.51** | Personal history of malignant neoplasm of bladder |
|  | |
|  | |
| **ICD10 Code** | **ICD10 Description** |
| **C67.0** | Malignant neoplasm of trigone of bladder |
| **C67.1** | Malignant neoplasm of dome of bladder |
| **C67.2** | Malignant neoplasm of lateral wall of bladder |
| **C67.3** | Malignant neoplasm of anterior wall of bladder |
| **C67.4** | Malignant neoplasm of posterior wall of bladder |
| **C67.5** | Malignant neoplasm of bladder neck |
| **C67.6** | Malignant neoplasm of ureteric orifice |
| **C67.7** | Malignant neoplasm of urachus |
| **C67.8** | Malignant neoplasm of overlapping sites of bladder |
| **C67.9** | Malignant neoplasm of bladder, unspecified |
| **D09.0** | Carcinoma in situ of bladder |
| **D41.4** | Neoplasm of uncertain behavior of bladder |
| **D49.4** | Neoplasm of unspecified behavior of bladder |
| **Z85.50** | Personal history of malignant neoplasm of unspecified urinary tract organ |
| **Z85.51** | Personal history of malignant neoplasm of bladder |
|  | |
